# Comparative analysis of alfalfa (*Medicago sativa* L.) leaf transcriptomes reveals genotype-specific salt tolerance mechanisms

**DOI:** 10.1186/s12870-018-1250-4

**Published:** 2018-02-15

**Authors:** Yunting Lei, Yuxing Xu, Christian Hettenhausen, Chengkai Lu, Guojing Shen, Cuiping Zhang, Jing Li, Juan Song, Honghui Lin, Jianqiang Wu

**Affiliations:** 1 Ministry of Education Key Laboratory for Bio-Resource and Eco-Environment, College of Life Science, State Key Laboratory of Hydraulics and Mountain River Engineering, Sichuan University, Chengdu, 610000 China; 2 Department of Economic Plants and Biotechnology, Yunnan Key Laboratory for Wild Plant Resources, Kunming Institute of Botany, Chinese Academy of Sciences, Kunming, 650201 China

**Keywords:** Alfalfa, *Medicago sativa*, Salt stress, Abscisic acid, Constitutive expression

## Abstract

**Background:**

Soil salinity is an important factor affecting growth, development, and productivity of almost all land plants, including the forage crop alfalfa (*Medicago sativa*). However, little is known about how alfalfa responds and adapts to salt stress, particularly among different salt-tolerant cultivars.

**Results:**

Among seven alfalfa cultivars, we found that Zhongmu-1 (ZM) is relatively salt-tolerant and Xingjiang Daye (XJ) is salt-sensitive. Compared to XJ, ZM showed slower growth under low-salt conditions, but exhibited stronger tolerance to salt stress. RNA-seq analysis revealed 2237 and 1125 differentially expressed genes (DEGs) between ZM and XJ in the presence and absence of salt stress, among which many genes are involved in stress-related pathways. After salt treatment, compared with the controls, the number of DEGs in XJ (19373) was about four times of that in ZM (4833). We also detected specific differential gene expression patterns: In response to salt stress, compared with XJ, ZM maintained relatively more stable expression levels of genes related to the ROS and Ca^2+^ pathways, phytohormone biosynthesis, and Na^+^/K^+^ transport. Notably, several salt resistance-associated genes always showed greater levels of expression in ZM than in XJ, including a transcription factor. Consistent with the suppression of plant growth resulting from salt stress, the expression of numerous photosynthesis- and growth hormone-related genes decreased more dramatically in XJ than in ZM. By contrast, the expression levels of photosynthetic genes were lower in ZM under low-salt conditions.

**Conclusions:**

Compared with XJ, ZM is a salt-tolerant alfalfa cultivar possessing specific regulatory mechanisms conferring exceptional salt tolerance, likely by maintaining high transcript levels of abiotic and biotic stress resistance-related genes. Our results suggest that maintaining this specific physiological status and/or plant adaptation to salt stress most likely arises by inhibition of plant growth in ZM through plant hormone interactions. This study identifies new candidate genes that may regulate alfalfa tolerance to salt stress and increases the understanding of the genetic basis for salt tolerance.

**Electronic supplementary material:**

The online version of this article (10.1186/s12870-018-1250-4) contains supplementary material, which is available to authorized users.

## Background

Soil salinization affects more than 800 million hectares of irrigated land and is a significant factor limiting agricultural productivity worldwide [1]. Breeding salt-tolerant crop varieties is therefore critical for the usage of these saline areas. Even though various traits have been identified as having an association with salinity tolerance in crops, including ion exclusion, osmotic tolerance, and tissue tolerance [2], a more comprehensive understanding of how plants respond to high salinity is still needed to facilitate the breeding of salt-tolerant crops.

High salt levels cause ion toxicity (mainly Na^+^), hyperosmotic stress, and secondary stresses such as oxidative damage [3]. Na^+^ stress triggers an increase in cytosolic Ca^2+^, and thereafter, Ca^2+^-binding proteins further activate downstream pathways [2]. At the same time, other second messengers linked to Ca^2+^ signaling, such as reactive oxygen species (ROS), are also induced [4]. Although ROS act as signaling molecules [5], high levels of ROS also result in oxidative damage and cell death in plants subjected to salt stress; thus the dynamic changes of enzyme activities related to ROS production and scavenging, are required for salt stress adaption [6]. In addition, stress-responsive plant hormones such as ABA also play an essential role in salt stress tolerance [7, 8]. Activated Ca^2+^, ROS, and phytohormone signaling cascades further alter plant transcriptomes by regulating transcription factors (TFs) such as AP2/ERFs, WRKYs, and bZIPs, causing changes in the expression of various genes [2], such as *HKT* and *NHX* gene families that contribute to plant salt-tolerance [9].

Alfalfa (*Medicago sativa* L.) is widely used as perennial legume forage due to its high protein content and biomass production [10]. Compared with many other crops, alfalfa is relatively tolerant to salt stress [9]. However, soil salinity is still an important environmental factor limiting yield in alfalfa. Selection of salinity-tolerant alfalfa germplasm has been attempted via a variety of approaches. Recently, several genes involved in salt tolerance have been isolated and characterized in alfalfa, including TFs [11–13], miRNAs [14], genes related to the biosynthesis of metabolites [15, 16], and other abiotic stress resistance-associated genes [17–20]. Moreover, the rapidly developing analytical chemistry technologies, transcriptomic [14, 21–23], proteomic [24–26], and genome-wide association analyses [27], have become important tools to dissect the mechanisms underlying alfalfa responses to salt stress.

Although only roots are directly exposed to the saline soil environment, leaves are also important for adaptation to high salinity. In response to salt stress, the growth of young leaves is inhibited and senescence of mature leaves is accelerated [9]. Furthermore, the growth of shoots is more arrested by salt stress than that of roots, and salt can build up in leaves to excessive levels [9]. It has been suggested that some salt-responsive genes might function in the sequestration of Na^+^ into leaf vacuoles, or the excretion of Na^+^ via special structures such as salt glands [9, 28]. In alfalfa, although both the roots and whole seedlings have been studied for their responses to salt stress, little is known about how leaves adapt to salinity through changes at the physiological and molecular level. Furthermore, previous studies have mainly focused on the germination and seedling establishment stages, and the ways in which mature-stage alfalfa responds to salt stress have not been studied in detail [29, 30].

Based on the growth phenotypes under salt treatment conditions, we selected two cultivars from seven alfalfas, the relatively salt-tolerant alfalfa Zhongmu-1 (ZM) and the salt-sensitive alfalfa Xinjiang Daye (XJ), and we analyzed the responses of mature-stage ZM and XJ to salt stress at both physiological and transcriptional levels. Transcriptomic analysis indicated abundant abiotic and biotic stress resistance-related differentially expressed genes (DEGs) between ZM and XJ in the absence and presence of salt stress. The expression levels of many salt-responsive genes, including TFs, were higher in ZM than in XJ, even under low-salt conditions. These candidate genes can be further analyzed for their functions in alfalfa salt tolerance and used for genetic engineering or breeding new salt-tolerant cultivars.

## Methods

### Plant materials and salt treatment

Alfalfa (*Medicago sativa*) seeds of seven cultivars (cvs. Zhongmu-1, Longdong, Hexi, Sandli, Eureka, Tianshui and Xingjiang Daye) were kindly provided by Prof. Quanwen Dou (Northwest Institute of Plateau Biology, Chinese Academy of Sciences, Xining, China). Among these, Zhongmu-1 was bred for being saline- and alkaline-tolerant in the laboratory of Dr. Qingchuan Yang in 1997, and has been widely cultivated as a salt-tolerant cultivar [23]; Xingjiang Daye is an alfalfa cultivar originally from Xinjiang province, which is adapted to the local cold and dry weather. Xingjiang Daye is relatively salt-sensitive at the mature stage [30], but salt-tolerant at the germination and seedling stage [31]. Alfalfa seeds were germinated on wet sterile filter paper in Petri dishes, and the seedlings were transferred to 1-L pots with soil 5 days after germination. Plants were cultivated in a greenhouse with a 20 to 28 °C temperature range and 16-h-light/8-h-dark cycle. When the plants were 30 days old, they were each watered with 500 mL of either water (the control group) or 0.5 M NaCl solution (the treatment group) once, and thereafter all plants were watered with water regularly to keep normal soil moisture. Seven days after treatment, the third leaves from the top were collected, frozen in liquid nitrogen and stored at − 80 °C. Twenty days after treatment, the aboveground dry masses were weighed; 2 months after treatment, the survival rates were calculated. Furthermore, the growth phenotypes were recorded 20, 30, and 40 days after treatment.

### Measurement of physiological and biochemical indexes

The total chlorophyll was extracted with acetone from fresh leaves and measured spectrophotometrically following the method described by Arnon et al. [32]. Relative water content (RWC) was measured according to Barrs and Weatherly [33]. Malondialdehyde (MDA) levels were assessed by determining thiobarbituric acid (TBA) reactive substances [34]. Superoxide levels were visually detected with nitro blue tetrazolium (NBT) as described previously [35]. Protein contents were estimated using the Bradford method and bovine serum albumin was used as the standard [36]. The activity of superoxide dismutase (SOD), peroxidase (POD), ascorbate peroxidase (APX), and catalase (CAT) were determined as described previously [37–40].

### Quantification of phytohormones

Phytohormone determination was done following Wu et al. [41]. In brief, about 150 mg of frozen leaf tissue was ground in liquid nitrogen, and 1 mL of ethyl acetate spiked with the internal standards D_6_-ABA, D_4_-SA, and D_5_-JA was added to each sample. Samples were vortexed for 10 min, and then centrifuged at 13,000 g for 10 min at 4 °C. The supernatants were transferred into fresh tubes and evaporated to dryness in a vacuum concentrator (Eppendorf, Germany). Each residue was resuspended in 0.4 mL of 70% methanol (*v*/v) and centrifuged at 13,000 g for 10 min at 4 °C to remove particles. Supernatants were transferred to glass vials and hormone measurements were carried out on an LCMS-8040 (Shimadzu, Japan) equipped with a Shim-pack XR-ODS column (2.0 × 75 mm, 2.2 μm) (Shimadzu). The column temperature was set at 40 °C and the flow rate was 0.27 mL/min.

### RNA isolation and RNA-seq analysis

Three biological replicates from each cultivar and treatment were used for RNA-seq analysis. Total RNA was isolated from leaf samples using the Trizol reagent (Invitrogen, USA) according to the manufacturer’s instructions. RNA quality and quantity were determined with a spectrophotometer (IMPLEN, Germany) and an Agilent 2100 Bioanalyzer (Agilent Technologies, USA).

RNA-seq was performed at the Novogene Company (Beijing, China). The RNA-Seq library was constructed using the NEBNext® Ultra™ RNA Library Prep Kit for Illumina® (NEB, USA). The mRNA was purified from total RNA using poly-T oligo-attached magnetic beads. The cleaved RNA fragments were transcribed into first-strand cDNA using reverse transcriptase and subsequently second-strand cDNA synthesis was performed using DNA polymerase I and RNase H. The fragments were ligated to sequencing adaptors and the library preparations were sequenced on an Illumina HiSeq 4000 platform and 150 bp paired-end reads were generated.

### De novo assembly and functional annotation

Raw reads were cleaned by removing adapters and low-quality sequences (reads with ambiguous bases ‘N’ and reads with more than 10% Q < 20 bases) with Cutadapt (https://cutadapt.readthedocs.io/en/stable/) and Btrim [42]. The cleaned reads were mapped to the reference genome of *M. truncatula* (Mt 4.0), but the mapping ratio was lower than 50%, probably due to the highly heterozygous genome of *M. sativa*, and because *M. sativa* is an autotetraploid [43]. Thus, de novo assembly of the transcriptomes was performed using the Trinity (v2.1.1) software [44]. Based on the Trinity assembly results, the gene functions were annotated using the Trinotate pipeline (http://trinotate.github.io/) with BLAST and HMMER, which provided information from UniProtKB/Swiss-Prot, Gene Ontology (GO), Kyoto Encyclopedia of Genes and Genomes (KEGG) databases, Pfam database, and Eggnog database using BLAST. In addition, Trinity assembly results were annotated with reference to Mt. 4.0.

### Differential expression analysis

The cleaned reads were mapped to the assembled sequences using Bowite2 [45], and for a specific transcript, the mapped reads were counted and the abundance was estimated using the RSEM method in the Trinity transcript quantification pipeline to obtain the FPKM, TPM, and expected count. The differential expression between two samples was identified using the Trinity differential expression pipeline in the DESeq2 package [46]. The *p* values were obtained from a differential gene expression test. FDR manipulation was used to determine the *p* value in multiple tests and analyses. Both a FDR < 0.05 and the absolute value of the Log_2_ (fold change) ≥ 1 were used as the threshold to identify genes with significantly different levels of expression.

## Results

### Growth status in the presence and absence of salt stress

**Fig. 1:**
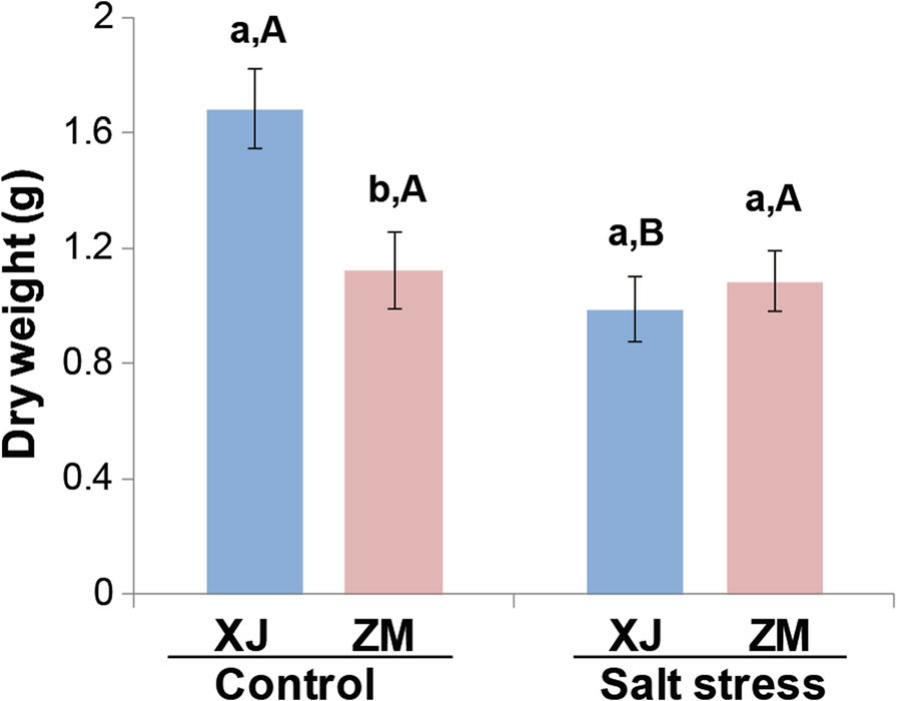
Biomass differences between XJ and ZM in response to salt stress. Alfalfa XJ and ZM plants (30 days old) were treated with 0.5 M NaCl (salt stress) or kept in normal soil (control). After 20 days, the shoot of every plant was harvested, and the aboveground biomass was measured (*n* = 50). All data are shown as mean ± standard error. Different lowercase letters represent significant differences between cultivars; different uppercase letters indicate significant differences between treatments (Tukey HSD test; *P* < 0.05)

Comparing seven alfalfa cultivars 2 months after 500 mL of 0.5 M NaCl treatment, we found that ZM had the highest survival rate (85%) and showed a strong salt-tolerant phenotype (Additional file 1: Figure S1). XJ was identified as the most salt-sensitive (35% survived) compared with the other six cultivars (Additional file 1: Figure S1). Therefore, ZM and XJ were chosen for comparing their growth under mid-and-long term salt stress. One month old XJ and ZM plants were treated with 500 mL of either water (control group) or 0.5 M NaCl solution (treatment group). Twenty days after treatment, under the control conditions, the above-ground biomass of ZM was 33% lower than that of XJ (Fig. 1); salt stress more strongly suppressed the growth of XJ than that of ZM (Additional file 1: Figure S2a), resulting in 41% decreased biomass, but salt treatment did not have a significant effect on the biomass of ZM (Fig. 1). After 30 days, when XJ started to wither, ZM showed moderate suppression of growth (Additional file 1: Figure S2b). After 40 days, the XJ plants had died, whereas the ZM plants survived with around 50% of dead leaves (Additional file 1: Figure S2c).

### Physiological differences between ZM and XJ cultivars in response to salinity stress

**Fig. 2:**
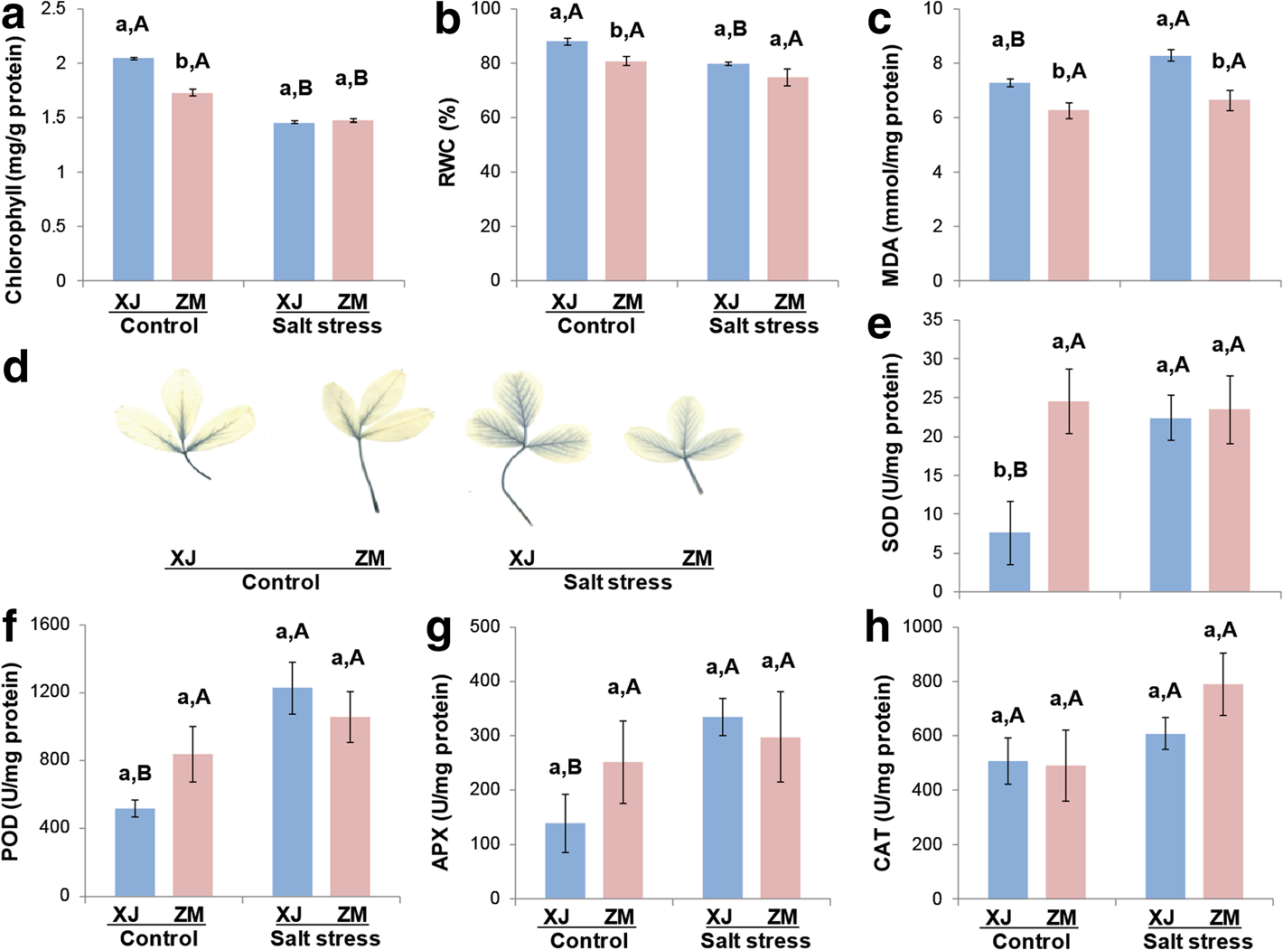
Salt-induced physiological changes in the leaves of XJ and ZM. XJ and ZM plants, about 30 days old, were treated with 0.5 M NaCl (salt stress) or kept in low-salt soil (control). After 1 week, leaf chlorophyll content (**a**), RWC (**b**), and MDA content (**c**) were determined, superoxide levels were visually detected by NBT staining (**d**), and the activity of antioxidant enzymes SOD (**e**), POD (**f**), APX (**g**), and CAT (**h**) were also measured. For chlorophyll, RWC, MDA, and superoxide level analysis, three replicates were used, and the activity of SOD, POD, APX, and CAT was determined from five replicates. All data are shown as mean ± standard error. Different lowercase letters represent significant differences between cultivars; different uppercase letters indicate significant differences between treatments (Tukey HSD test; *P* < 0.05)

Under the control condition, compared with ZM, XJ showed higher leaf chlorophyll content, relative water content (RWC), and malondialdehyde (MDA) level (Fig. 2a-c). One week after salt stress, the chlorophyll content and RWC of XJ were decreased and MDA content was elevated, but these metrics appeared to have little or no change in ZM (Fig. 2a-c). Notably, MDA content remained at lower levels in ZM than in XJ (Fig. 2c).

One week after salt stress, compared to ZM, XJ plants accumulated higher levels of superoxide than did the control plants (Fig. 2d). In the absence of salt, the SOD activity in ZM was 2.24-fold greater than in XJ and did not change after salt treatment (Fig. 2e); in contrast, salt stress strongly enhanced the SOD activity in XJ to a level that is similar to the activity of SOD in ZM (Fig. 2e). Furthermore, similar patterns of POD and APX activity were also detected in ZM and XJ (Fig. 2f-g). However, CAT activity was similar between ZM and XJ and was not significantly induced after salt stress in either cultivar (Fig. 2h).

Taken together, these results indicate that the salt-sensitive XJ responded to salinity stress with high levels of ROS and elevation of ROS-related enzyme activity, whereas the salt-tolerant ZM showed relatively lower levels of ROS production and unaltered activity of ROS-related enzymes.

### Transcriptome sequencing of ZM and XJ cultivars

Samples for RNA-seq were collected on day seven after treating ZM and XJ with salt solution (0.5 M NaCl) (for simplicity, in the treatment group, ZM and XJ are designated as TZM and TXJ, respectively; in the control group, ZM and XJ are designated as CZM and CXJ, respectively). In total, 226,165,617 clean reads were obtained from the 12 RNA-seq datasets, and 555,014 unigenes (≥ 200 bp), with a mean length of 418 bp, were de novo assembled using the Trinity assembly software (V2.1.1) (Additional file 1: Figure S3; Additional file 2: Table S1). For functional annotation, the sequences of the assembled unigenes were compared to a variety of databases and 383,958 unigenes were annotated with putative functions based on hits from at least one database (Additional file 2: Table S2).

### Overall identification and functional annotation of differentially expressed genes

**Fig. 3:**
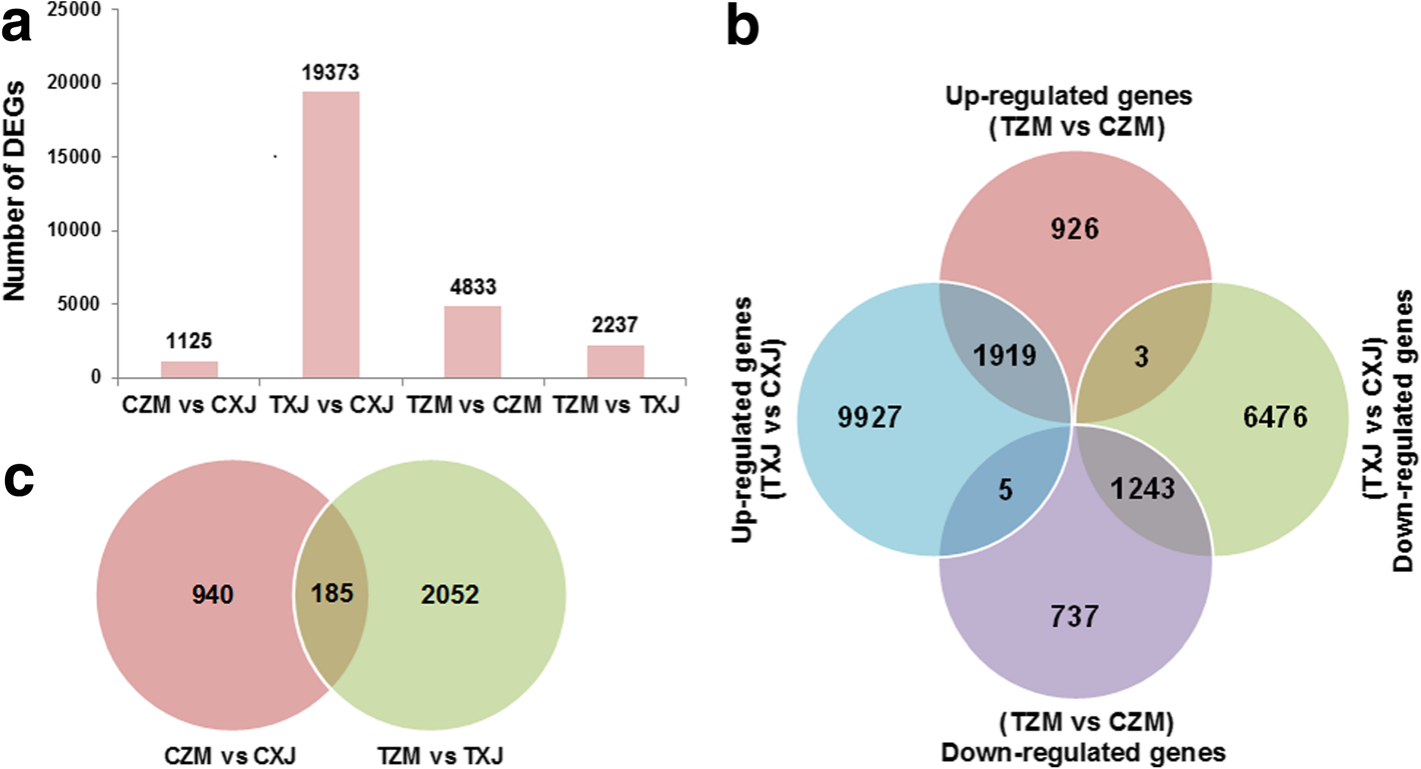
DEGs in XJ and ZM in the presence and absence of salt stress. XJ and ZM were treated with salt solution (treatment group; TXJ and TZM, respectively) or water (control group; CZM and CXJ, respectively), and subsequently samples were collected on the seventh day. **a** Summary of the number of DEGs in the presence and absence of salt stress. **b** Venn diagram indicating the DEGs from comparisons between CZM and CXJ and between TZM and TXJ. **c** Euler diagram of salt-responsive genes, including up- and down-regulated genes, in XJ and ZM

The differentially regulated genes of ZM and XJ in response to salt stress were analyzed in the RNA-seq datasets. In the control group, 1125 genes were differentially expressed between ZM and XJ (CZM vs CXJ) (Fig. 3a; Additional file 2: Table S3), and GO and KEGG analyses revealed that many of these DEGs are involved in abiotic and biotic stress response-related pathways, such as “response to stimulus” (127 genes), “immune system process” (9 genes), and “plant-pathogen interaction” (4 genes) (Additional file 1: Figure S4a-b). In samples collected 7 days after the salt treatment, 2237 DEGs were identified between ZM and XJ (TZM vs TXJ) (Fig. 3a; Additional file 2: Table S3), and these DEGs might ultimately be the cause of the differences between these alfalfa cultivars’ adaption to salt stress. Many of these DEGs were annotated with the GO biological process terms “response to stimulus”, “reactive oxygen species”, “responding to stress”, “response to hormone”, and other stress-responsive processes (Additional file 1: Figure S4c). To explore the biological pathways important for alfalfa responses to salt stress, the DEGs were further annotated to the reference pathways in KEGG where a large number of them were mapped to the pathways including hormone signal transduction, plant-pathogen interaction, peroxisome, and biosynthesis of secondary metabolites (such as phenylpropanoid biosynthesis) (Additional file 1: Figure S4d). Furthermore, Venn diagram revealed that 185 genes were always differentially expressed between ZM and XJ, regardless of the presence or absence of salt stress (Fig. 3b).

To identify genes potentially involved in salt resistance, we further analyzed the salt-responsive DEGs in both cultivars. Compared with their respective control samples (TXJ vs CXJ and TZM vs CZM), 19,373 (11,651 up- and 7722 down-regulated) and 4833 (2848 up- and 1985 down-regulated) DEGs were found in salt-treated XJ and ZM, respectively (Fig. 3a; Additional file 2: Table S3). Thus, about three times more salt-responsive genes were differentially regulated in XJ than in ZM. Comparison of GO and KEGG annotations between these two cultivars showed that the salt-responsive gene clusters/pathways enriched in XJ were similar to those in ZM (Additional file 1: Figure S4a-b). Euler diagram of these DEGs (Fig. 3c; Additional file 2: Table S3) indicated that in response to salt treatment: 1) 1663 (926 up- and 737 down-regulated) DEGs were specifically regulated in ZM, while over 16,000 (9927 up- and 6476 down-regulated) DEGs were specifically regulated in XJ; 2) 1919 and 1243 genes were co-up- or co-down-regulated in ZM and XJ compared to their respective controls; 3) 3 and 5 genes were up- and down-regulated in ZM but were down- and up-regulated in XJ, compared to their respective controls.

### Differentially regulated genes involved in ROS homeostasis and Ca^2+^ signaling

**Fig. 4:**
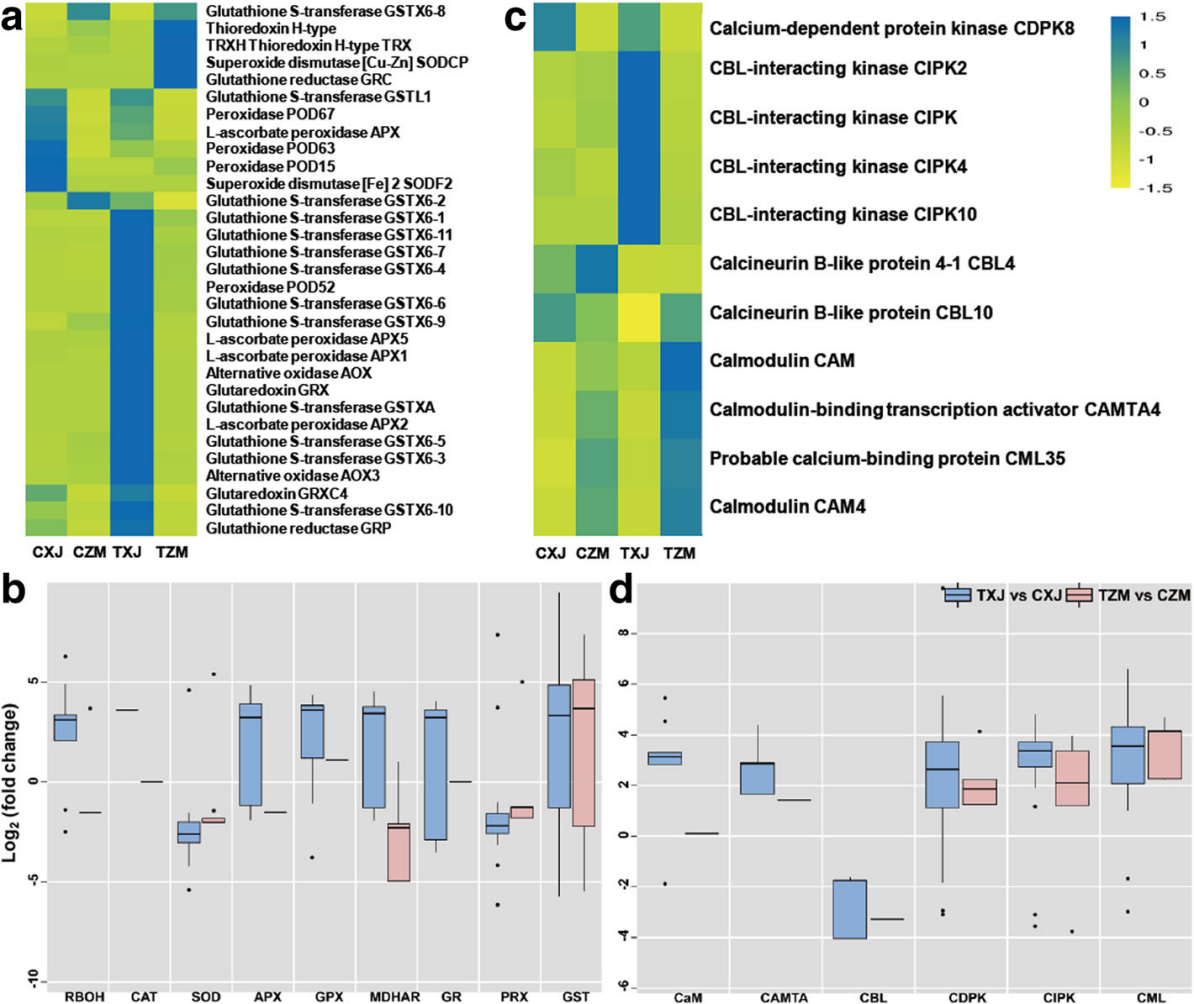
Expression of genes involved in second messenger signaling (ROS and Ca^2+^) in XJ and ZM. XJ and ZM were treated with salt solution (TXJ and TZM, respectively) or water (CZM and CXJ, respectively), and subsequently samples were collected on the seventh day. **a** Heatmap of the relative expression of the genes important for ROS catabolism. **b** Box plot indicating the expression changes of the genes involved in ROS homeostasis, in XJ and ZM, in response to salt treatment. **c** Heatmap of the relative expression of the genes important for Ca^2+^ downstream signaling. **d** Box plot indicating the expression changes of the salt-responsive genes involved in Ca^2+^ downstream signaling in XJ and ZM. Further detailed information is given in Additional file 2: Tables S4 and S5

Surprisingly, except for a few genes belonging to the *SOD*, *TRX*, and *GR* families, most genes involved in ROS scavenging showed lower expression levels in the salt-tolerant ZM than in the salt-sensitive XJ in the absence and presence of salt stress, including *POD*s, *GST*s, *APX*s, *GRX*s, and *AOX*s (Fig. 4a; Additional file 2: Table S4). Perhaps the low levels of ROS in ZM do not require high activity of ROS scavenging enzymes; thus, these ROS scavenging enzyme genes showed low transcript levels. In response to salt stress, the genes encoding RBOH proteins, known as plant enzymatic ROS-generating systems, were induced or suppressed in both cultivars; however, *RBOH*s in XJ were more strongly elevated than in ZM (Fig. 4b; Additional file 2: Table S4). Moreover, certain salt-responsive genes involved in ROS scavenging systems, including *CAT*s, *SOD*s*, APX*s, *GPX*s, *MDHAR*s, *GR*s, *PRX*s, and *GST*s, were strongly upregulated in XJ but only slightly changed in ZM (Fig. 4b; Additional file 2: Table S4).

Salt stress-induced Ca^2+^ signaling likely plays an important role in plant adaptation to salt stress. In the absence and presence of salt stress, some genes encoding proteins of the CAM, CMATA, and CML families were expressed at higher levels in ZM than in XJ, whereas the expression levels of *CDPK*s and *CIPK*s were at lower levels in ZM than in XJ (Fig. 4c; Additional file 2: Table S5). Notably, *CBL4* (*SOS3*), which is an important calcium sensor in plant salt tolerance [47], had higher expression levels in ZM than in XJ under control conditions, but decreased to similar levels under salt stress conditions (Fig. 4c; Additional file 2: Table S5). In response to salt stress, except for *CBLs*, which showed decreased levels, most Ca^2+^-trigger downstream genes were upregulated in both cultivars, including *CAM*s, *CAMAT*s, *CDPK*s, *CIPK*s and *CML*s (Fig. 4d; Additional file 2: Table S5), and compared with those in XJ, salt-induced *CAM*s, *CAMAT*s, and *CIPK*s were less strongly affected on the expression levels in ZM (Fig. 4d; Additional file 2: Table S5).

### Phytohormone levels and DEGs involved in phytohormone biosynthesis

**Fig. 5:**
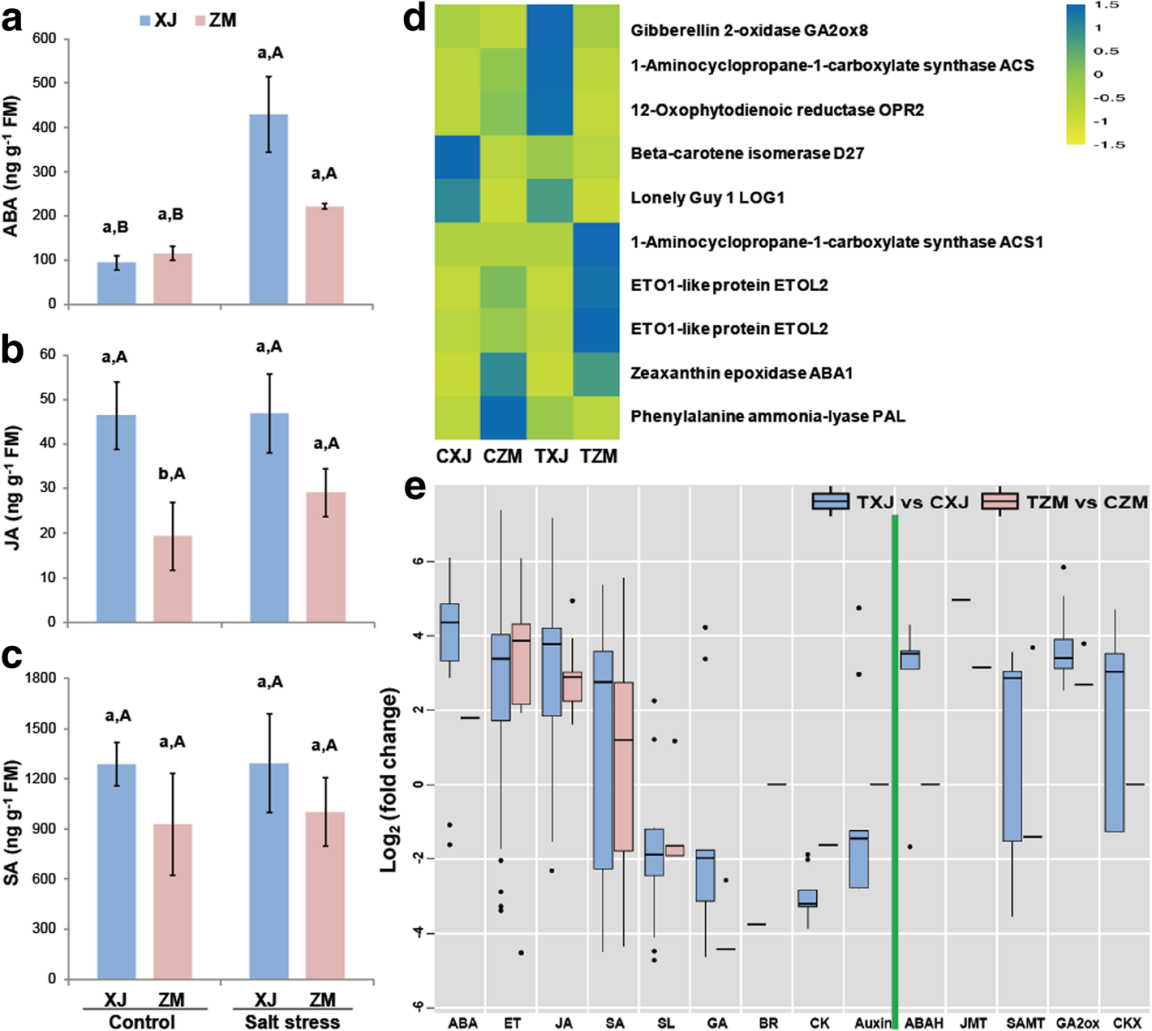
ABA, JA, and SA levels and expression of phytohormone biosynthetic genes in XJ and ZM. XJ and ZM were treated with salt solution (TXJ and TZM, respectively) or water (CZM and CXJ, respectively), and subsequently samples were collected on the seventh day. ABA (**a**), JA (**b**), and SA (**c**) levels in ZM and XJ cultivars (*n* = 5). All data are shown as mean ± standard error. Different lowercase letters represent significant differences between cultivars; different uppercase letters indicate significant differences between treatments (Tukey’s HSD test; *P* < 0.05). **d** Heatmap of the relative expression of the genes encoding phytohormone biosynthesis enzymes. **e** Box plot indicating the expression changes of the salt-responsive genes involved in phytohormone biosynthesis in XJ and ZM. Further detailed information is given in Additional file 2: Table S6

Salt stress increased the ABA content of XJ 3.55-fold, whereas ZM only exhibited 92% elevated ABA content (Fig. 5a). The concentration of JA was not altered by salt treatment in XJ, whereas it was increased by 50.8% in ZM; furthermore, the JA content was always greater in XJ than in ZM (Fig. 5b). We did not detect different levels of SA between these two alfalfa cultivars under control or salt stress conditions (Fig. 5c).

Next, we inspected the DEGs involved in plant hormone biosynthesis between ZM and XJ in the absence and presence of salt stress. The ethylene biosynthetic genes *ACS*s did not show a consistent pattern in either alfalfa cultivar (Fig. 5d; Additional file 2: Table S6). Nevertheless, two *ETOL2*s, which encode negative post-transcriptional regulators of ACS [48], showed higher levels of expression in ZM than in XJ following salt stress (Fig. 5d; Additional file 2: Table S6). In the absence and presence of salt stress, the ABA and SA biosynthetic genes *ABA1* and *PAL* were at higher level in ZM than in XJ, respectively; in contrast, the expression level of *GA2ox*, which is involved in gibberellin (GA) catabolism, was lower in ZM than in XJ with and without salt stress (Fig. 5d; Additional file 2: Table S6). Similarly, *OPR2*, *D27*, and *LOG1*, which are involved in JA, strigolactone (SL), and cytokinin (CK) biosynthesis, had also lower levels of expression in ZM than in XJ, respectively (Fig. 5d; Additional file 2: Table S6).

The salt stress-induced and suppressed genes in ZM and XJ involved in phytohormone biosynthesis, were also examined. Most of the ABA biosynthetic genes, such as *ABA1*, *ABA2*, and *NCED*s, displayed enhanced expression in XJ but not in ZM (Fig. 5e; Additional file 2: Table S6). Strikingly, the expression levels of *ABAH*s*/CYP707A*s, which are related to ABA catabolism, were enhanced at least 8-fold in XJ, but not in ZM (Fig. 5e; Additional file 2: Table S6). In both cultivars, the genes involved in the ET and JA biosynthesis were also enhanced despite lack of changes in the JA contents (Fig. 5e; Additional file 2: Table S6). In contrast to ABA, the contents of SA and the expression of its biosynthetic genes were not significantly enhanced or decreased (Fig. 5e; Additional file 2: Table S6). Expression levels of most salt-responsive SL, GA, and CK biosynthetic genes showed decreases in both alfalfa cultivars (Fig. 5e; Additional file 2: Table S6). Moreover, the levels of the brassinosteroid (BR) and auxin biosynthetic genes were decreased in XJ but unchanged in ZM after salt-stress treatment (Fig. 5e; Additional file 2: Table S6). The expression levels of *JMT*, *SAMT*, *GA2ox*, and *CKX*, which are involved in JA, SA, GA, and CK catabolism respectively, showed less change in ZM than in XJ cultivars after salt treatment (Fig. 5e; Additional file 2: Table S6).

Thus, in response to salt stress, the salt-susceptible XJ increased ABA-, ET-, and JA-related transcripts, but decreased the expression of SL, GA, BR, CK, and auxin biosynthetic genes involved in growth. By contrast, ZM exhibited relatively smaller changes in the levels of these genes, particularly the ABA-related transcripts (Fig. 5e; Additional file 2: Table S6). This is consistent with the growth phenotypes following salt treatment, in which XJ was arrested in growth but ZM was only slightly influenced.

### DEGs encoding Na^+^/K^+^ transport proteins for ion homeostasis

**Fig. 6:**
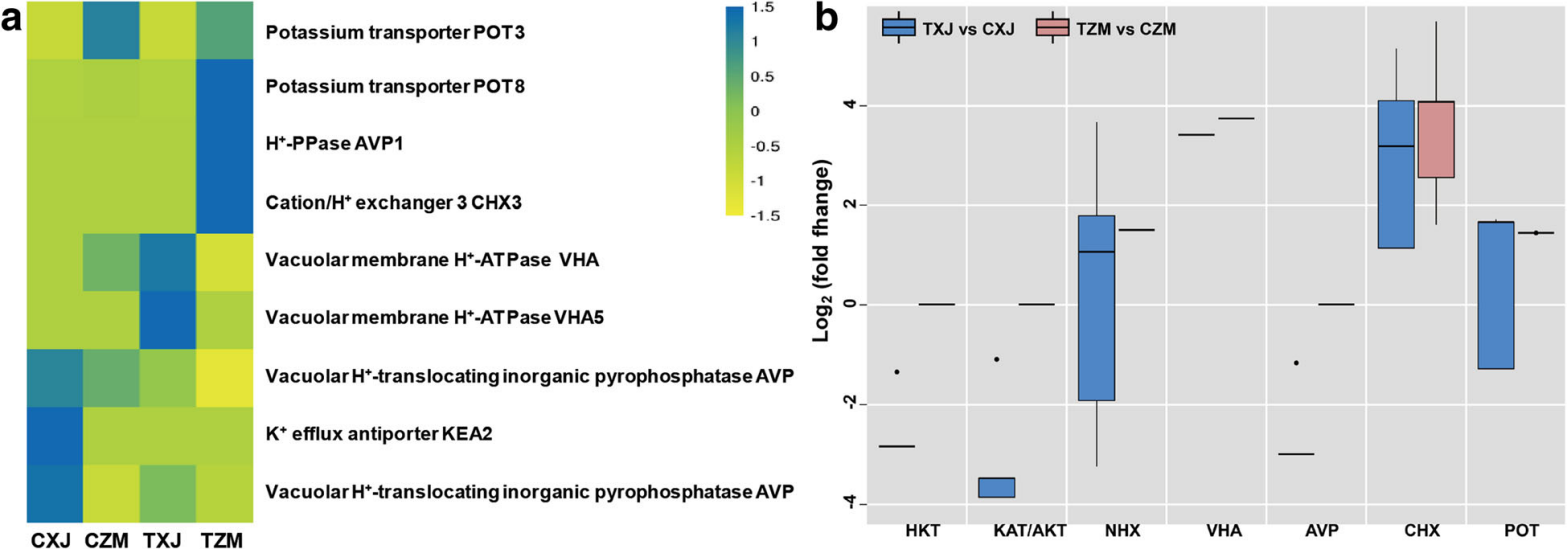
Expression of the genes involved in ion transport in XJ and ZM. XJ and ZM were treated with salt solution (TXJ and TZM, respectively) or water (CZM and CXJ, respectively), and subsequently samples were collected on the seventh day. **a** Heatmap of the relative expression levels of genes encoding ion transporters. **b** Box plot indicating the expression changes of the salt-responsive genes of ion transporters in XJ and ZM. Further detailed information is given in Additional file 2: Table S7

Our analysis also revealed DEGs related to ion transporters (especially Na^+^/K^+^ transporters) which are important for ion homeostasis (Fig. 6a; Additional file 2: Table S7). Under salt-stress conditions, *POT8*, *AVP1*, and *CHX3* were expressed at higher levels in ZM than in XJ; nevertheless, two *VHA*s had lower levels of expression in ZM than in XJ (Fig. 6a; Additional file 2: Table S7). Notably, the levels of *POT3* and two *AVP*s were always higher and lower in ZM than in XJ, respectively (Fig. 6a; Additional file 2: Table S7).

In response to salt stress, the genes encoding HKT, AKT/KAT, and AVP families, which are essential Na^+^ transporters that mitigate elevated Na^+^ concentrations [49], were hardly changed in ZM, but were strongly suppressed in XJ (Fig. 5b; Additional file 2: Table S7). Why these Na^+^ transporter genes were down-regulated in XJ remains unclear. Other genes encoding Na^+^/K^+^ transporters, including CHX, CNGC, NHX, PM, and VHA, were enhanced or suppressed by salt stress in both alfalfa cultivars but had no significant difference in expression levels between ZM and XJ (Fig. 6b; Additional file 2: Table S7). These results suggest that ZM and XJ might have very different strategies to regulate cytoplasmic Na^+^ levels and maintain ion homeostasis in response to salt stress.

### DEGs encoding transcription factors

**Fig. 7:**
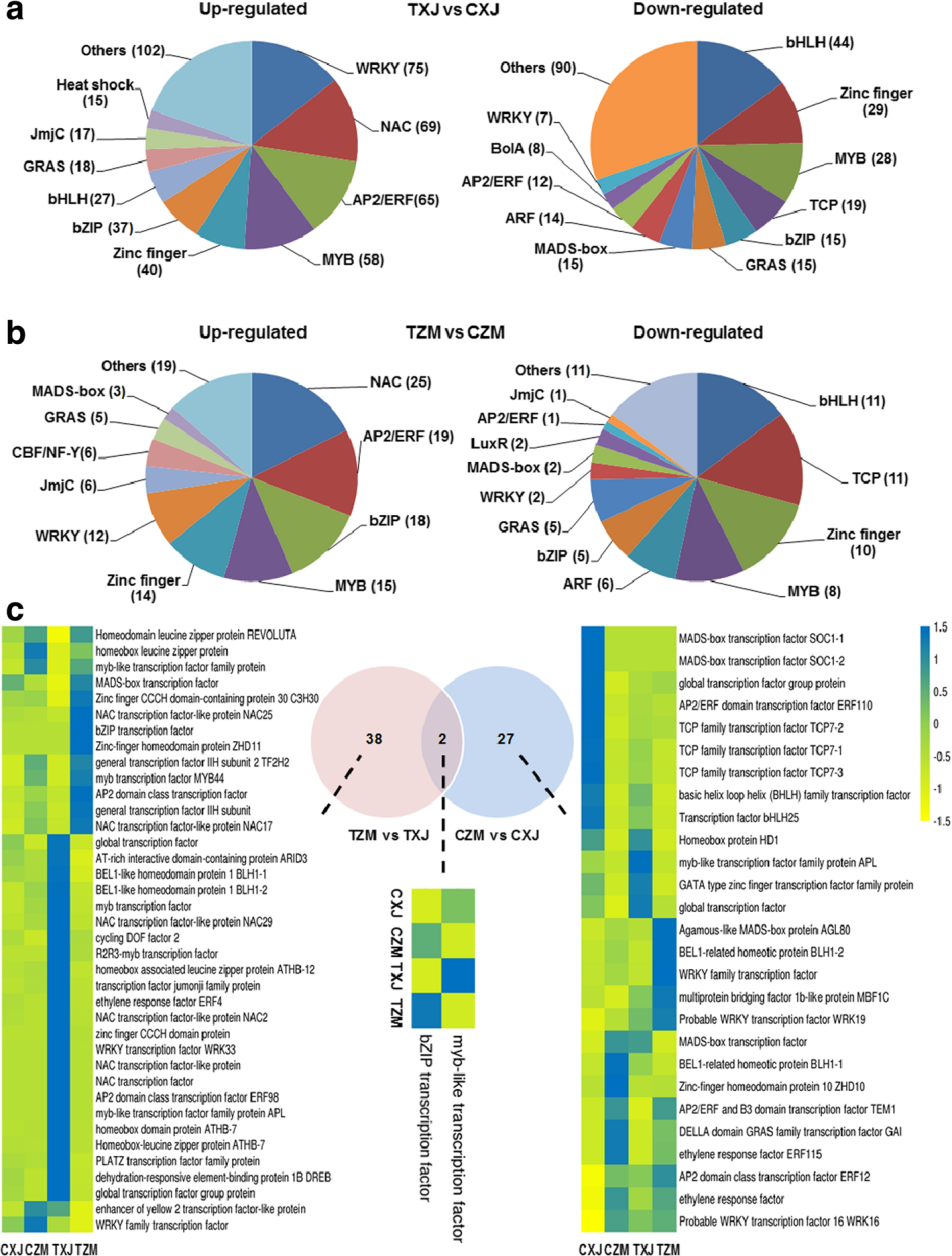
Expression of the genes encoding transcription factors in XJ and ZM. XJ and ZM were treated with salt solution (TXJ and TZM, respectively) or water (control group; CZM and CXJ, respectively), and subsequently samples were collected on the seventh day. In response to salt treatment, the up- and down-regulated transcription factor gene families (gene numbers are shown in the brackets) in XJ (**a**) and ZM (**b**) were identified with RNA-seq analysis. **c** Venn diagram showing the numbers of DEGs encoding transcription factors between ZM and XJ under control and salt treatment conditions. Heatmaps indicate the relative gene expression levels of the DEGs. Further detailed information is given in Additional file 2: Table S8

Salt stress induced or suppressed many more TFs in XJ than in ZM: 158 and 78 TF genes were up- and down-regulated in ZM, while 533 and 298 were up- and down-regulated in XJ, respectively (Fig. 7a-b; Additional file 2: Table S8). Furthermore, WRKY, NAC, AP2/ERF, MYB, Zinc finger, and bZIP were the top six most upregulated TF families, while bHLH, TCP, MYB, and Zinc finger were the top four most downregulated TF families in these two cultivars (Fig. 7a-b; Additional file 2: Table S8).

In the control group, comparing XJ and ZM, 29 differentially expressed transcription factors (DETFs) were detected; after salt treatment, there were 40 DETFs between XJ and ZM. Among all these DETFs, two TFs (a bZIP and a MYB) always exhibited greater or lower levels of expression in ZM than in XJ (Fig. 7c; Additional file 2: Table S8), regardless of salt stress or control conditions. It is possible that these TFs could function in regulating plant adaptation to salt stress and partly account for the salt tolerance of ZM.

### Gene expression related to plant photosynthesis

**Fig. 8:**
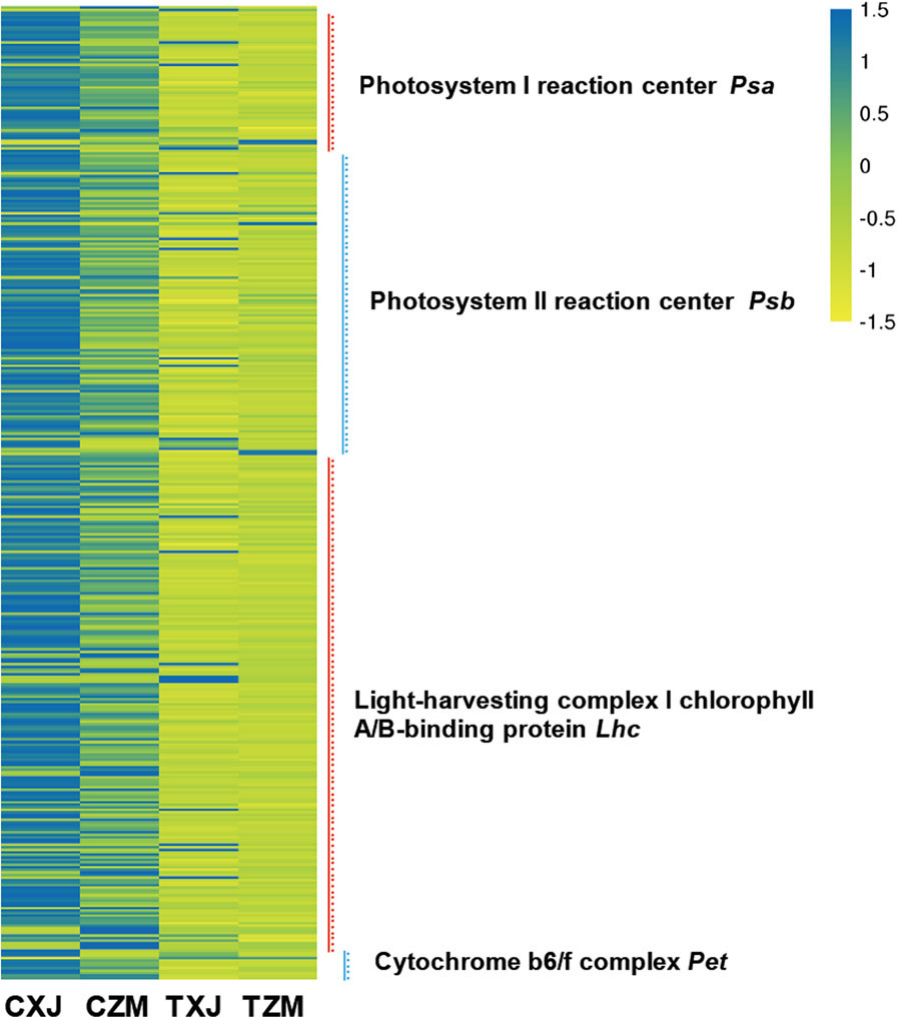
Comparison of photosynthesis-related genes in XJ and ZM under low-salt and salt-stress conditions. XJ and ZM were treated with salt solution (TXJ and TZM, respectively) or water (control group; CZM and CXJ, respectively), and subsequently samples were collected on the seventh day. Heatmap indicates the relative transcript levels of the genes from four genes families, *Lhc*s, *Pet*s, *Psa*s and *Psb*s, which are important for photosynthesis. Further detailed information is given in Additional file 2: Table S9

Expression patterns of the genes involved in plant growth, such as photosynthesis-related genes, also differed between these alfalfa cultivars. In the plants of the control group, most of the photosynthetic genes, such as *Lhc*s, *Pet*s, *Psa*s, and *Psb*s, expressed at lower levels in ZM than in XJ (Fig. 8; Additional file 2: Table S9). Following salt treatment, most of photosynthetic genes were repressed in both alfalfa cultivars, whereas the expression levels decreased more strongly in XJ than in ZM and the expression levels of many photosynthetic genes were not significantly different between these two alfalfa cultivars anymore (Fig. 8; Additional file 2: Table S9).

## Discussion

In wild species, genetic diversity is the driving force behind the adaptation to local environments. In crops, cultivars with diverse genetic backgrounds are important for breeding new varieties with improved agronomic traits. It is known that alfalfa cultivars which are not sensitive to salt during the germination or seedling stage may be sensitive to salt during the later vegetative growth [10]. Thus, studying the responses of mature-stage alfalfa plants to salt stress may provide additional important insight into the mechanisms underlying alfalfa salt tolerance. Here, we therefore utilized two alfalfa varieties ZM and XJ at the mature stage and explored how their leaves respond to salinity. In this study, we found that at the mature stage, ZM likely uses a constitutive salt-resistance strategy at the cost of relatively slow growth, while XJ uses an inducible strategy but overall cannot adapt so well to salt stress.

### Transcriptional variation in adaptation to salinity exist in different alfalfa tissues

Although many pathways involved in plant responses to salt stress may be conserved in most plants, their relative importance may vary with species, varieties, and even tissues [9]. For instance, salt-stress rapidly induced more ABA accumulation in maize roots than in leaves [50]. Following salinity stress, the expression levels of most genes regulating ABA biosynthesis in leaves were increased in both alfalfas (Fig. 5e; Additional file 2: Table S6). This is in contrast to the ABA receptor genes *PYL6*s*,* which were down-regulated in alfalfa roots [22]. Sodium-proton exchangers, such as plasma and vacuolar membrane Na^+^/H^+^ exchangers, are key regulation factors that maintain low cytoplasmic Na^+^ concentrations in plant cells [2, 7]. Among most of the 12 alfalfa genotypes subjected to salt stress, *SOS1* (encoding a plasma membrane Na^+^/H^+^ exchanger) is expressed at higher levels in root than in leaf tissue [51]. However, in our leaf transcriptomics experiments in both alfalfa cultivars, some of *CHX*/*VHAs* encoding vacuolar membrane Na^+^/H^+^ exchanger, rather than *SOS1*, were induced to very high levels in response to salinity stress (Fig. 6b; Additional file 2: Table S7), which supports the notion that these plants prevent excessive cytosolic Na^+^ accumulation by compartmentalizing Na^+^ into vacuoles via the corresponding vacuolar Na^+^/H^+^ exchangers [52].

Some of the ERF TFs are involved in plant resistance to salinity [11, 12, 53]. Our results show that in response to salt stress, among all the differentially regulated *AP2/ERF*s in XJ and ZM, 65 (77%) and 12 (92%) *AP2/ERF*s were up-regulated, respectively (Fig. 7; Additional file 2: Table S8). However, Postnikova et al. found that a majority of salt-responsive *AP2/ERF*s were down-regulated in alfalfa roots [22]. Furthermore, the expression levels of *RD22*s (mediated by ABA) in alfalfa roots were enhanced by salt stress [22]; nevertheless, we found that most *RD22*s were suppressed by salt stress in our alfalfa leaves (Additional file 2: Table S3). Stress-responsive genes *ERD*s and *DRPE*s were down-regulated in alfalfa root [22], but most of these genes were up-regulated in ZM and XJ leaves (Additional file 2: Table S3). These results support the idea that variations in transcriptional regulation during adaptation to salt stress exist in different tissues of alfalfa.

### Phytohormone interactions for trade-off between salt-resistance and growth in alfalfa

Plant hormones including GAs, BRs, auxin, CKs, and SLs are central for the regulation of plant growth and development [54–56]. Under low-salt conditions, XJ grew faster than did ZM, and the above-ground biomass of XJ was greater (Fig. 1); consistently, the genes *D27* and *LOG1* (CK and SL biosynthetic genes, respectively) were more highly expressed in XJ (Fig. 5d; Additional file 2: Table S6). Following salt treatment, however, most of the growth-related phytohormone biosynthetic genes were repressed following salt stress, especially in XJ (Fig. 5e; Additional file 2: Table S6) and this is in agreement with the growth of these two cultivars (Fig. 1). Furthermore, salt stress down-regulates photosynthetic genes, due to the combined effects of dehydration and osmotic stress [57]. Under low-salt conditions, ZM grew slower than did XJ (Fig. 1); accordingly, most of photosynthesis-related genes were expressed at lower levels in ZM than in XJ (Fig. 8; Additional file 2: Table S9). After salt treatment, plant growth was inhibited and most of the photosynthetic genes were highly down-regulated in XJ, while ZM was not affected in growth and exhibited moderate down-regulation of photosynthesis-related genes (Figs. 1 and 7; Additional file 2: Table S9). Recent studies also have shown that plant hormones affect photosynthesis in response to different abiotic stress conditions [58]. Furthermore, salt-triggered pathways increase the levels of growth-repressing DELLAs, at least partly through a reduction in the contents of bioactive GAs [59]. Therefore, it is likely that in alfalfa, salt-stress responses (such as growth inhibition) are regulated at least in part by changed levels of plant hormones.

ABA content in plants exposed to drought or high salinity increases dramatically, inducing stress-tolerance effects that help plants to adapt and survive under stress conditions [60]. Many phytohormones also function in inhibiting plant growth during stress adaptation [61], such as ABA’s inhibition of the BR and GA pathway [62, 63]. Following salt treatment, although the levels of ABA were observed to increase in both alfalfa cultivars, XJ showed a greater increase than did ZM (Fig. 5a). This was consistent with the stronger growth arrest in XJ (Fig. 1). Therefore, the results suggest that phytohormone interactions may play a part in the trade-off between salt-resistance and growth during alfalfa adaptation to salt stress.

### Diverging strategies regulating resistance to salt stress in alfalfa cultivars

Previously, it was found that in many alfalfa cultivars, salt treatment led to decreased chlorophyll content and RWC, and these were higher in the salt-tolerant cultivars than in the salt-sensitive ones [26, 30, 64, 65]; in contrast, MDA were increased, and these were lower in the salt-tolerant cultivars than in more salt-sensitive ones [26, 31, 66]. In this study, following salt stress, a similar effect was detected in XJ but not in ZM except for the chlorophyll content (Fig. 2a-c). Moreover, the activity of antioxidant enzymes SOD, APX, and POD increased in XJ after salt treatment and reached similar levels to those in ZM, and these enzymes’ activity did not change in ZM (Fig. 2e-g). Compared with XJ, the growth phenotype of ZM and its relatively small changes in enzymatic activity suggest that ZM likely uses a constitutive resistance strategy to adapt to salt stress, while XJ responses to salt stress in an inducible manner.

From the transcriptomic data, we found that several abiotic stress-related genes were constitutively expressed at higher levels in ZM than in XJ (Figs. 4, 5, 6 and 7; Additional file 2: Table S3). It was previously shown that the constitutive high expression of noted abiotic stress-related genes, such as *TEM1* [67], *MYB4* [68], *HDA6* [69], *NFD4* [70], and *ADF3* [71], which are known to confer salt tolerance to plants, were at higher levels in ZM than in XJ not only following salt stress but also under low-salt conditions (Additional file 2: Table S3). Therefore, it is likely that the constitutively overexpressed abiotic stress resistance-related genes at least partly account for the salt tolerance phenotype of ZM.

The regulatory factors shaping the differences between ZM and XJ in salt tolerance remain unclear. We found various genes involved in phytohormone pathways, ion, and ROS homeostasis to be differentially regulated between these two cultivars. Generally, XJ showed stronger changes in genes involved in these pathways, while in ZM they had much smaller alterations. Notably, a bZIP transcription factor was found to be constitutively expressed more highly in ZM than in XJ (Fig. 7). In Arabidopsis, a bZIP gene has been found to be a positive regulator of plant tolerance to salt, osmotic and drought stresses [72]. Further functional analyses are needed to confirm the role of this bZIP in alfalfa tolerance to salt stress.

Plants perceive salt stress through a yet unknown mechanism and rapidly activate Ca^2+^ messengers and ROS production [9]. Immediately thereafter, Ca^2+^ sensors (e.g., CBLs, CIPKs, and CDPKs, etc.), ROS signaling, and phytohormones (probably at least partly modulated by Ca^2+^ and ROS), further regulate transcriptome reconfiguration, increasing the plant’s capability to adapt to salt stress [2]. Given the large differences of the transcriptome profiles of ZM and XJ, it is plausible that a number of the upstream regulatory genes were selected during ZM breeding, leading to high salt-tolerance in ZM. More research, including using QTL mapping, is needed to identify the main genetic elements that contribute to the strong salt tolerance in ZM.

In the field, unlike our experimental setup, soil salt contents do not sudden increase. Plants have to adapt to salt conditions even starting from germination. Although ZM is more salt-tolerant than is XJ both under our lab conditions (sudden salt application) and in the field (ZM was bred for salt tolerance, while XJ was bred for cold and drought resistance), very likely these two cultivars use different mechanisms to adapt to the field saline soil from how they respond to sudden/short-term salt stress, and this should be further studied.

## Conclusions

Previously, changes in expression of salt-responsive genes among 12 alfalfa genotypes indicated that most stress tolerance genes were more dramatically upregulated in salt-tolerant genotypes compared to the sensitive ones [51]. Here, we found that the salt-tolerant ZM showed almost no changes in growth after salt treatment, while that of the salt-sensitive XJ was strongly arrested. Phytohormone quantification, enzyme activity assay, and transcriptomic analysis all suggested that ZM uses a constitutive strategy to adapt to salt stress, although with the cost of slower growth under low-salt conditions. Further reverse genetic analysis of the DEGs between ZM and XJ, especially the TFs, might further reveal the mechanisms underlying salt tolerance of ZM.

## Additional files

Additional file 1: This PDF contains all of the additional material (Figure S1–S5) associated with the manuscript. Figure numbers and titles are listed below: **Figure S1.** The survival rates of seven alfalfa cultivars. **Figure S2.** The growth phenotypes of XJ and ZM cultivars. **Figure S3.** Distribution of the lengths of the assembled unigenes in the transcriptomes of alfalfa leaf tissue. **Figure S4.** The GO and KEGG annotations of DEGs between XJ and ZM. **Figure S5.** The GO and KEGG annotations of the salt-responsive genes compared between XJ and ZM. (PDF 1351 kb)

Additional file 2: This excel file contains all of the additional Tables (Table S1-S9) associated with the manuscript. Each Table is in a different tab. Table numbers and titles are listed below: **Table S1.** Summary of clean reads and mapping ratio. **Table S2.** Number of annotated unigenes in alfalfa. **Table S3.** Annotations of DEGs in two cultivars of alfalfa. **Table S4.** The DEGs involved in ROS homeostasis in two cultivars. **Table S5.** The DEGs involved in Ca^2+^ signaling in two cultivars. **Table S6.** The DEGs involved in phytohormone biosynthesis in two cultivars**. Table S7.** The DEGs involved in ion transport in two cultivars**. Table S8.** The DEGs involved in transcription factor in two cultivars**. Table S9.** The photosynthesis-related DEGs in two cultivars. (XLSX 6789 kb)

## Abbreviations

ABA1: Zeaxanthin epoxidase
ABA2: Xanthoxin dehydrogenase
ABAH/CYP707A: ABA 8′-hydroxylase
ACS: 1-Aminocyclopropane-1- carboxylic acid synthase
ADF3: Cofilin/actin-depolymerizing factor-like protein 3
AKT/KAT: K^+^ inward rectifying channel
AOX: Alternative oxidase
APX: Ascorbate peroxidase
AVP: Vacuolar pyrophosphatase
CAM: Calmodulin
CAT: Catalase
CBL: Calcineurin B-like protein
CDPK: Calcium-dependent protein kinase
CHX: Cation-proton exchanger
CIPK: CBL-interacting kinase
CKX: Cytokinin oxidase/dehydrogenase
CMATA: Calmodulin-binding transcription activator
CML: Calmodulin-like protein
CNGC: Cyclic nucleotide-gated ion channel
D27: Dwarf 27
DRPE: Desiccation-related protein
ERD: Early-responsive to dehydration
ETOL: ETO1-like protein
FDR: False discovery rate
FPKM: Fragments Per Kilobase transcript length per Million fragments mapped
GA2ox: Gibberellin 2-oxidase
GR: Glutathione reductase
GRX: Glutaredoxin
GST: Glutathione S-transferase
HDA6: Histone deacetylase family protein 6
HKT: High-affinity K^+^ transporter
JMT: Jasmonic acid carboxyl methyltransferase
Lhc: Light-harvesting chlorophyll protein complex
LOG1: Lonely guy 1
MDHAR: Monodehydroascorbate reductase
NCED: 9-cis-epoxycarotenoid dioxygenase
NFD4: Nuclear fusion defective 4
NHX: Na^+^/K^+^ exchanger
OPR: 12-oxophytodienoate reductase
PAL: Phenylalanine-ammonia lyase
Pet: Cytochrome b6-f complex
PM: Plasma membrane H^+^-ATPase
POD: Peroxidase
POT: Potassium transporter
PRX: Peroxiredoxin
Psa: Photosystem I protein
Psb: Photosystem II protein
PYL: Pyrabactin resistance 1-like
QTL: Quantitative trait loci
RBOH: Respiratory burst oxidase homologue
RD22: Responsive to dehydration 22
SAMT: Salicylic acid carboxyl methyltransferase
SOD: Superoxide dismutase
SOS1: Salt over sensitive 1
TEM1: Tempranillo 1
TPM: Transcripts Per Million transcripts
TRX: Thioredoxin
VHA: vacuolar H^+^-translocating inorganic pyrophosphatase

## References

[1] Roy SJ, Negrao S, Tester M (2014). Salt resistant crop plants. Curr Opin Biotechnol.

[2] Deinlein U, Stephan AB, Horie T, Luo W, Xu G, Schroeder JI (2014). Plant salt-tolerance mechanisms. Trends Plant Sci.

[3] Zhu JK (2002). Salt and drought stress signal transduction in plants. Annu Rev Plant Biol.

[4] Steinhorst L, Kudla J (2013). Calcium and reactive oxygen species rule the waves of signaling. Plant Physiol.

[5] Kurusu T, Kuchitsu K, Tada Y (2015). Plant signaling networks involving Ca^2+^and Rboh/Nox-mediated ROS production under salinity stress. Front Plant Sci.

[6] Miller G, Suzuki N, Ciftci-Yilmaz S, Mittler R (2010). Reactive oxygen species homeostasis and signalling during drought and salinity stresses. Plant Cell Environ.

[7] Zhu JK (2016). Abiotic stress signaling and responses in plants. Cell.

[8] Sah SK, Reddy KR, Li J (2016). Abscisic acid and abiotic stress tolerance in crop plants. Front Plant Sci.

[9] Munns R, Tester M (2008). Mechanisms of salinity tolerance. Annu Rev Plant Biol.

[10] Scasta JD, Trostle CL, Foster MA (2012). Evaluating alfalfa (*Medicago sativa* L.) cultivars for salt tolerance using laboratory, greenhouse and field methods. J Agr Sci.

[11] Chen T, Yang Q, Zhang X, Ding W, Gruber M (2012). An alfalfa (*Medicago sativa* L.) ethylene response factor gene, *MsERF11*, enhances salt tolerance in transgenic Arabidopsis. Plant Cell Rep.

[12] Chen T, Yang Q, Gruber M, Kang J, Sun Y, Ding W (2012). Expression of an alfalfa (*Medicago sativa* L.) ethylene response factor gene *MsERF8* in tobacco plants enhances resistance to salinity. Mol Biol Rep.

[13] Winicov II, Bastola DR (1999). Transgenic overexpression of the transcription factor alfin1 enhances expression of the endogenous *MsPRP2* gene in alfalfa and improves salinity tolerance of the plants. Plant Physiol.

[14] Long RC, Li MN, Kang JM, Zhang TJ, Sun Y, Yang QC (2015). Small RNA deep sequencing identifies novel and salt-stress-regulated microRNAs from roots of *Medicago sativa* and *Medicago truncatula*. Physiol Plant.

[15] Palma F, Tejera NA, Lluch C (2013). Nodule carbohydrate metabolism and polyols involvement in the response of *Medicago sativa* to salt stress. Environ Exp Bot.

[16] Lai DW, Mao Y, Zhou H, Li F, Wu MZ, Zhang J (2014). Endogenous hydrogen sulfide enhances salt tolerance by coupling the reestablishment of redox homeostasis and preventing salt-induced K^+^ loss in seedlings of *Medicago sativa*. Plant Sci.

[17] Long RC, Zhang F, Li ZY, Li MN, Cong LL, Kang JM (2015). Isolation and functional characterization of salt-stress induced *RCI2*-like genes from Medicago sativa and *Medicago truncatula*. J Plant Res.

[18] Ginzberg I, Stein H, Kapulnik Y, Szabados L, Strizhov N, Schell J (1998). Isolation and characterization of two different cDNAs of delta1-pyrroline-5-carboxylate synthase in alfalfa, transcriptionally induced upon salt stress. Plant Mol Biol.

[19] Miller G, Stein H, Honig A, Kapulnik Y, Zilberstein A (2005). Responsive modes of *Medicago sativa* proline dehydrogenase genes during salt stress and recovery dictate free proline accumulation. Planta.

[20] Zhang Z, Wang Y, Chang L, Zhang T, An J, Liu Y (2015). *MsZEP*, a novel zeaxanthin epoxidase gene from alfalfa (*Medicago sativa*), confers drought and salt tolerance in transgenic tobacco. Plant Cell Rep.

[21] An YM, Song LL, Liu YR, Shu YJ, Guo CH (2016). *De novo* transcriptional analysis of alfalfa inresponse to saline-alkaline stress. Front Plant Sci.

[22] Postnikova OA, Shao J, Nemchinov LG (2013). Analysis of the alfalfa root transcriptome in response to salinity stress. Plant Cell Physiol.

[23] Jin H, Sun Y, Yang Q, Chao Y, Kang J, Jin H (2010). Screening of genes induced by salt stress from alfalfa. Mol Biol Rep.

[24] Long R, Li M, Zhang T, Kang J, Sun Y, Cong L (2016). Comparative proteomic analysis reveals differential root proteins in *Medicago sativa* and *Medicago truncatula* in response to salt stress. Front Plant Sci.

[25] Ma QL, Kang JM, Long RC, Cui YJ, Zhang TJ, Xiong JB (2016). Proteomic analysis of salt and osmotic-drought stress in alfalfa seedlings. J Integr Agr.

[26] Rahman MA, Alam I, Kim YG, Ahn NY, Heo SH, Lee DG (2015). Screening for salt-responsive proteins in two contrasting alfalfa cultivars using a comparative proteome approach. Plant Physiol Bioch.

[27] Yu LX, Liu XC, Boge W, Liu XP (2016). Genome-wide association study identifies loci for salt tolerance during germination in autotetraploid alfalfa (*Medicago sativa* L.) using genotyping-by-sequencing. Front. Plant Sci.

[28] Yuan F, Lyu MJ, Leng BY, Zheng GY, Feng ZT, Li PH (2015). Comparative transcriptome analysis of developmental stages of the *Limonium bicolor* leaf generates insights into salt gland differentiation. Plant Cell Environ.

[29] Smith SE, Johnson DW, Conta DM, Hotchkiss JR (1994). Using climatological, geographical, and taxonomic information to identify sources of mature-plant salt tolerance in alfalfa. Crop Sci.

[30] Quan WL, Liu X, Wang HQ, Chan ZL (2016). Physiological and transcriptional responses of contrasting alfalfa (*Medicago sativa* L.) varieties to salt stress. Plant Cell Tiss Org.

[31] Wang WB, Kim YH, Lee HS, Kim KY, Deng XP, Kwak SS (2009). Analysis of antioxidant enzyme activity during germination of alfalfa under salt and drought stresses. Plant Physiol Bioch.

[32] Arnon DI (1949). Copper enzymes in isolated chloroplasts. Polyphenoloxidase in *Bta vulgaris*. Plant Physiol.

[33] Barrs H, Weatherley P (1962). A re-examination of the relative turgidity technique for estimating water defiits in leaves. Aust J Biol Sci.

[34] Heath RL, Packer L (1968). Photoperoxidation in isolated chloroplasts. I. Kinetics and stoichiometry of fatty acid peroxidation. Arch Biochem Biophys.

[35] Jabs T, Dietrich RA, Dangl JL (1996). Initiation of runaway cell death in an Arabidopsis mutant by extracellular superoxide. Science.

[36] Bradford MM (1976). A rapid and sensitive method for the quantitation of microgram quantities of protein utilizing the principle of protein-dye binding. Anal Biochem.

[37] Giannopolitis CN, Ries SK (1977). Superoxide dismutases: I. Occurrence in higher plants. Plant Physiol.

[38] Aebi H (1984). Catalase in vitro. Methods Enzymol.

[39] Nakano Y, Asada K (1981). Hydrogen peroxide is scavenged by ascorbate specific peroxidase in spinach chloroplasts. Plant Cell Physiol.

[40] Zaharieva T, Yamashita K, Matsumoto H (1999). Iron deficiency induced changes in ascorbate content and enzyme activities related to ascorbate metabolism in cucumber roots. Plant Cell Physiol.

[41] Wu J, Hettenhausen C, Meldau S, Baldwin IT (2007). Herbivory rapidly activates MAPK signaling in attacked and unattacked leaf regions but not between leaves of *Nicotiana attenuata*. Plant Cell.

[42] Trapnell C, Roberts A, Goff L, Pertea G, Kim D, Kelley DR (2012). Differential gene and transcript expression analysis of RNA-seq experiments with TopHat and Cufflinks. Nat Protoc.

[43] Yu F, Wang H, Zhao Y, Liu R, Dou Q, Dong J (2017). Karyotypic evolution of the *Medicago* complex: *sativa-caerulea-falcata* inferred from comparative cytogenetic analysis. BMC Evol Biol.

[44] Haas BJ, Papanicolaou A, Yassour M, Grabherr M, Blood PD, Bowden J (2013). *De novo* transcript sequence reconstruction from RNA-seq using the trinity platform for reference generation and analysis. Nat Protoc.

[45] Langmead B, Salzberg SL (2012). Fast gapped-read alignment with Bowtie 2. Nat Methods.

[46] Love MI, Huber W, Anders S (2014). Moderated estimation of fold change and dispersion for RNA-seq data with DESeq2. Genome Biol.

[47] Halfter U, Ishitani M, Zhu JK (2000). The Arabidopsis SOS2 protein kinase physically interacts with and is activated by the calcium-binding protein SOS3. Proc Natl Acad Sci U S A.

[48] Christians MJ, Gingerich DJ, Hansen M, Binder BM, Kieber JJ, Vierstra RD (2009). The BTB ubiquitin ligases ETO1, EOL1 and EOL2 act collectively to regulate ethylene biosynthesis in Arabidopsis by controlling type-2 ACC synthase levels. Plant J.

[49] Maathuis FJ, Ahmad I, Patishtan J (2014). Regulation of Na^+^ fluxes in plants. Front Plant Sci.

[50] Jia WS, Wang YQ, Zhang SQ, Zhang JH (2002). Salt-stress-induced ABA accumulation is more sensitively triggered in roots than in shoots. J Exp Bot.

[51] Sandhu D, Cornacchione MV, Ferreira JF, Suarez DL (2017). Variable salinity responses of 12 alfalfa genotypes and comparative expression analyses of salt-response genes. Sci Rep.

[52] Ding M, Hou P, Shen X, Wang M, Deng S, Sun J (2010). Salt-induced expression of genes related to Na^+^/K^+^ and ROS homeostasis in leaves of salt-resistant and salt-sensitive poplar species. Plant Mol Biol.

[53] Bahieldin A, Atef A, Edris S, Gadalla NO, Ali HM, Hassan SM (2016). Ethylene responsive transcription factor ERF109 retards PCD and improves salt tolerance in plant. BMC Plant Biol.

[54] Depuydt S, Hardtke CS (2011). Hormone signalling crosstalk in plant growth regulation. Curr Biol.

[55] Zubo YO, Blakley IC, Yamburenko MV, Worthen JM, Street IH, Franco-Zorrilla JM (2017). Cytokinin induces genome-wide binding of the type-B response regulator ARR10 to regulate growth and development in Arabidopsis. Proc Natl Acad Sci U S A.

[56] Liu J, Novero M, Charnikhova T, Ferrandino A, Schubert A, Ruyter-Spira C (2013). *Carotenoid cleavage dioxygenase 7* modulates plant growth, reproduction, senescence, and determinate nodulation in the model legume *Lotus japonicus*. J Exp Bot.

[57] Chaves MM, Flexas J, Pinheiro C (2009). Photosynthesis under drought and salt stress: regulation mechanisms from whole plant to cell. Ann Bot.

[58] Peleg Z, Blumwald E (2011). Hormone balance and abiotic stress tolerance in crop plants. Curr Opin Plant Biol.

[59] Achard P, Cheng H, De Grauwe L, Decat J, Schoutteten H, Moritz T (2006). Integration of plant responses to environmentally activated phytohormonal signals. Science.

[60] Ng LM, Melcher K, Teh BT, Xu HE (2014). Abscisic acid perception and signaling: structural mechanisms and applications. Acta Pharmacol Sin.

[61] Nguyen D, Rieu I, Mariani C, van Dam NM (2016). How plants handle multiple stresses: hormonal interactions underlying responses to abiotic stress and insect herbivory. Plant Mol Biol.

[62] Clouse SD (2016). Brassinosteroid/abscisic acid antagonism in balancing growth and stress. Dev Cell.

[63] Razem FA, Baron K, Hill RD (2006). Turning on gibberellin and abscisic acid signaling. Curr Opin Plant Biol.

[64] Peng YL, Gao ZW, Gao Y, Liu GF, Sheng LX, Wang DL (2008). Eco-physiological characteristics of alfalfa seedlings in response to various mixed salt-alkaline stresses. J Integr Plant Biol.

[65] Anower MR, Mott IW, Peel MD, Wu Y (2013). Characterization of physiological responses of two alfalfa half-sib families with improved salt tolerance. Plant Physiol Bioch.

[66] Ashrafi E, Razmjoo J, Zahedi M, Pessarakli M (2015). Screening alfalfa for salt tolerance based onl ipid peroxidation and antioxidant enzymes. Agron J.

[67] Fu M, Kang HK, Son SH, Kim SK, Nam KH (2014). A subset of Arabidopsis RAV transcription factors modulates drought and salt stress responses independent of ABA. Plant Cell Physiol.

[68] Vannini C, Iriti M, Bracale M, Locatelli F, Faoro F, Croce P (2006). The ectopic expression of the rice *Osmyb4* gene in Arabidopsis increases tolerance to abiotic, environmental and biotic stresses. Physiol Mol Plant P.

[69] Chen LT, Luo M, Wang YY, Wu K (2010). Involvement of Arabidopsis histone deacetylase HDA6 in ABA and salt stress response. J Exp Bot.

[70] Sottosanto JB, Saranga Y, Blumwald E (2007). Impact of AtNHX1, a vacuolar Na^+^/H^+^ antiporter, upon gene expression during short- and long-term salt stress in *Arabidopsis thaliana*. BMC Plant Biol.

[71] Huang YC, Huang WL, Hong CY, Lur HS, Chang MC (2012). Comprehensive analysis of differentially expressed rice actin depolymerizing factor gene family and heterologous overexpression of *OsADF3* confers *Arabidopsis Thaliana* drought tolerance. Rice.

[72] Sun X, Li Y, Cai H, Bai X, Ji W, Ding X (2012). The Arabidopsis AtbZIP1 transcription factor is a positive regulator of plant tolerance to salt, osmotic and drought stresses. J Plant Res.

