# Supplementary material for: Comparative analysis of alfalfa (Medicago sativa L.) leaf transcriptomes reveals genotype-specific salt tolerance mechanisms
Source: BMC Plant Biol. 2018 Feb 15;18:35. doi: 10.1186/s12870-018-1250-4 (PMC5815232; doi:10.1186/s12870-018-1250-4)
Supplement: Supplementary file 1 — This PDF contains all of the additional material (Figure S1–S5) associated with the manuscript. Figure numbers and titles are listed below: Figure S1. The survival rates of seven alfalfa cultivars. Figure S2. The growth phenotypes of XJ and ZM cultivars. Figure S3. Distribution of the lengths of the assembled unigenes in the transcriptomes of alfalfa leaf tissue. Figure S4. The GO and KEGG annotations of DEGs between XJ and ZM. Figure S5. The GO and KEGG annotations of the salt-responsive genes compared between XJ and ZM. (PDF 1351 kb) [file 12870_2018_1250_MOESM1_ESM.pdf]

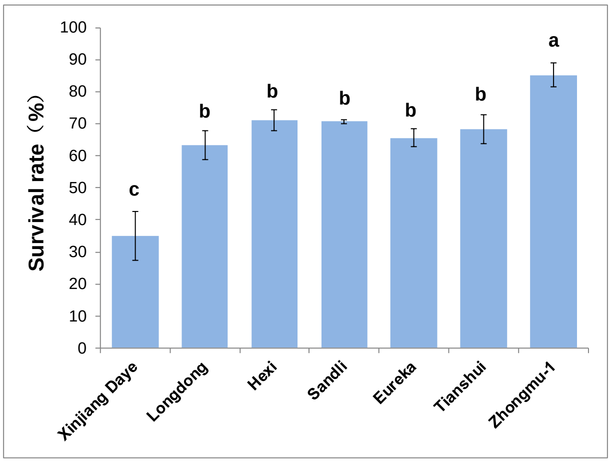

Figure S1 The survival rates of seven alfalfa cultivars.

Two months after salt treatment, the survival rates of seven alfalfa cultivars were counted (n=50). All data are shown as mean ±standard error. Different lowercase letters indicate significant differences among cultivars (Tukey' HSD test; $P < 0.05$).

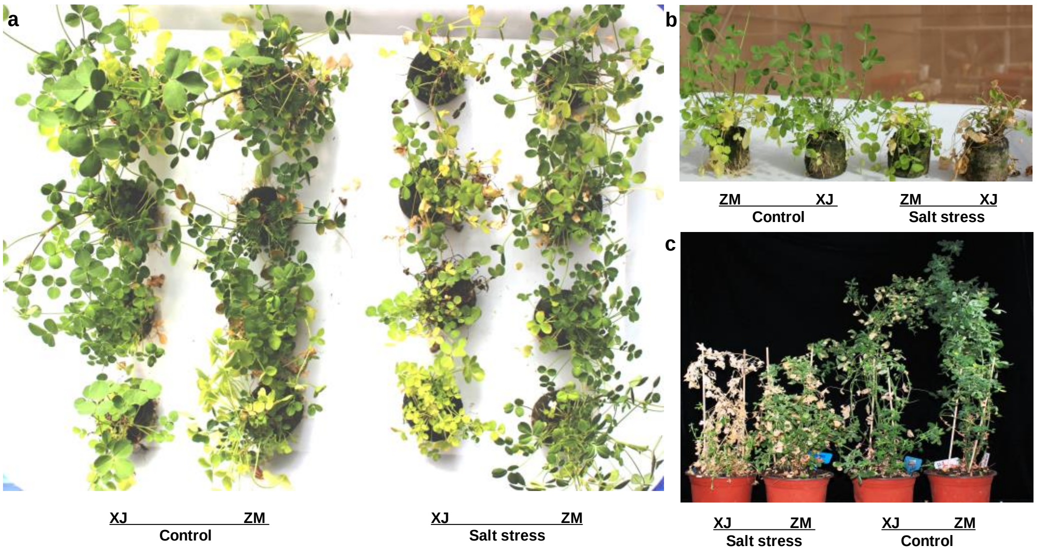

Figure S2 The growth phenotypes of XJ and ZM cultivars.

Thirty days old XJ and ZM plants, which were cultivated in seedling blocks or 1-L pots, were irrigated with water or 0.5 M NaCl solution. Thereafter, plants grown in seedling blocks were further cultivated for 20 days (**a**) or 30 days (**b**); plants cultivated in 1-L pots were further cultivated for 40 days (**c**). The growth phenotypes of the two cultivars were photographed.

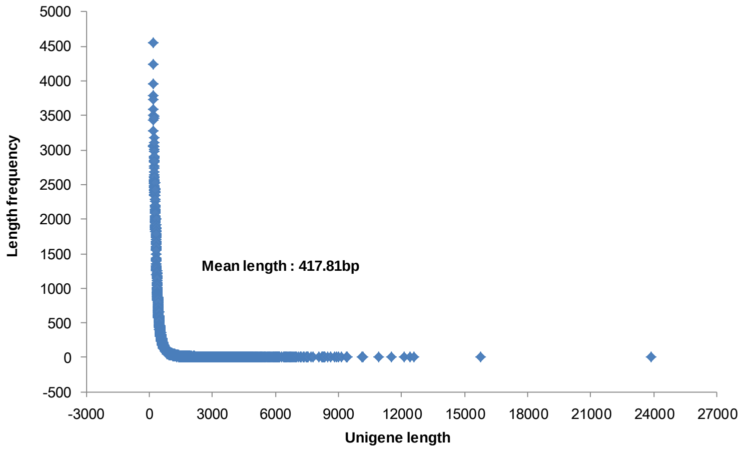

Figure S3 Distribution of the lengths of the assembled unigenes in the transcriptomes of alfalfa leaf tissue.

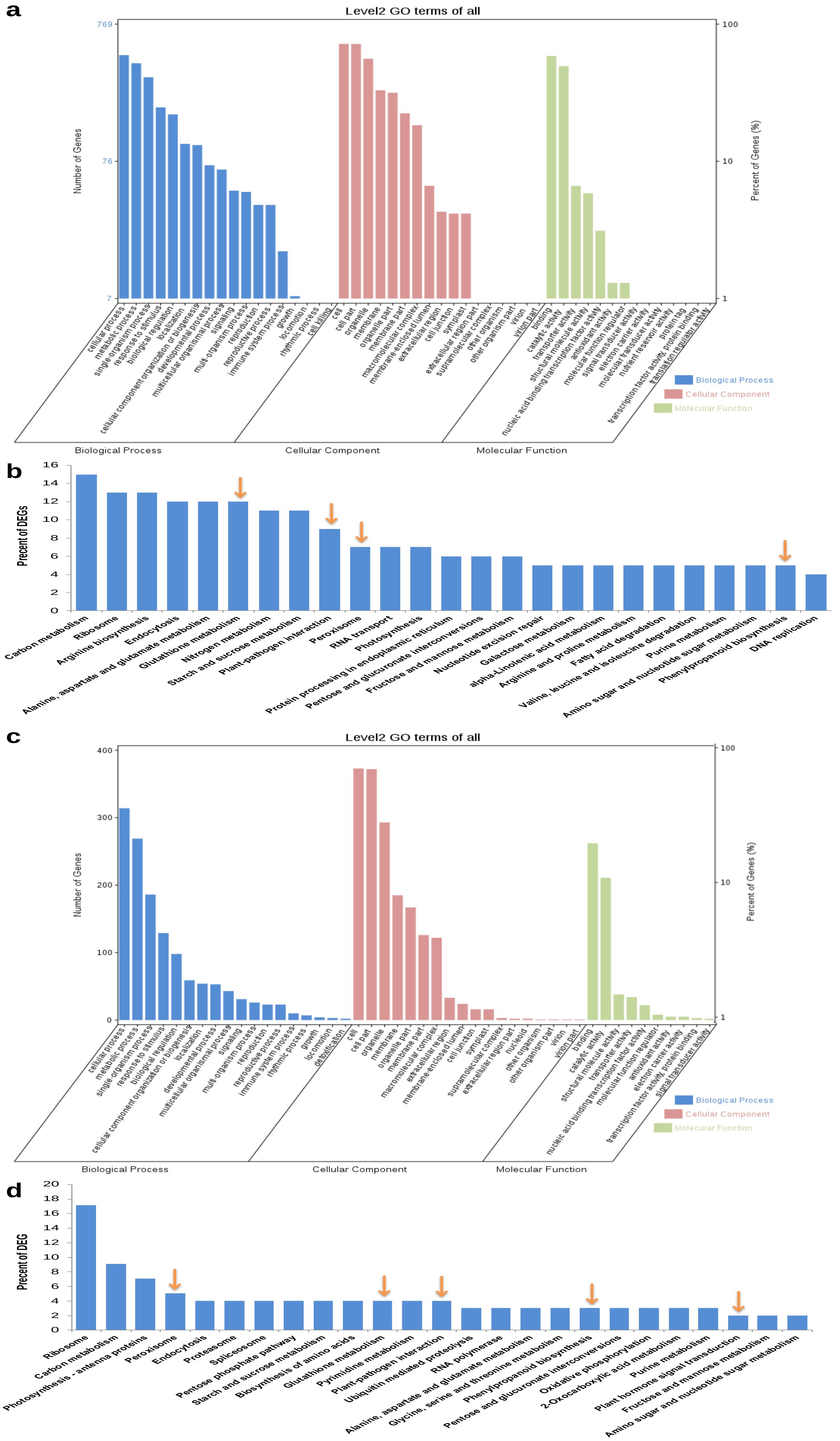
a
Level2 GO terms of all
769
76
7
Number of Genes
100
10
1
Percent of Genes (%)
cellular process
metabolic process
single-organism process
response to stimulus
biological regulation
localization
cellular component organization or biogenesis
developmental process
multicellular organismal process
signaling
multi-organism process
reproduction
reproductive process
immune system process
growth
locomotion
rhythmic process
cell killing
cell
cell part
organelle
membrane
organelle part
membrane part
macromolecular complex
membrane-enclosed lumen
extracellular region
cell junction
symplast
extracellular region part
supramolecular complex
other organism
other organism part
virion
virion part
binding
catalytic activity
transporter activity
structural molecule activity
nucleic acid binding transcription factor activity
antioxidant activity
molecular function regulator
signal transducer activity
electron carrier activity
molecular transducer activity
nutrient reservoir activity
protein tag
transcription factor activity, protein binding
translation regulator activity
Biological Process
Cellular Component
Molecular Function
b
16
14
12
10
8
6
4
2
0
Precent of DEGs
Carbon metabolism
Ribosome
Arginine biosynthesis
Endocytosis
Alanine, aspartate and glutamate metabolism
Glutathione metabolism
Nitrogen metabolism
Starch and sucrose metabolism
Plant-pathogen interaction
Peroxisome
RNA transport
Photosynthesis
Protein processing in endoplasmic reticulum
Pentose and glucuronate interconversions
Fructose and mannose metabolism
Nucleotide excision repair
Galactose metabolism
alpha-Linolenic acid metabolism
Arginine and proline metabolism
Fatty acid degradation
Valine, leucine and isoleucine degradation
Purine metabolism
Amino sugar and nucleotide sugar metabolism
Phenylpropanoid biosynthesis
DNA replication
c
Level2 GO terms of all
400
300
200
100
0
Number of Genes
100
10
1
Percent of Genes (%)
cellular process
metabolic process
single-organism process
response to stimulus
biological regulation
cellular component organization or biogenesis
localization
developmental process
multicellular organismal process
signaling
multi-organism process
reproduction
reproductive process
immune system process
rhythmic process
growth
locomotion
detoxification
cell
cell part
organelle
membrane
organelle part
membrane part
macromolecular complex
extracellular region
membrane-enclosed lumen
cell junction
symplast
supramolecular complex
extracellular region part
nucleoid
other organism
other organism part
virion
virion part
binding
catalytic activity
structural molecule activity
transporter activity
nucleic acid binding transcription factor activity
molecular function regulator
antioxidant activity
electron carrier activity
transcription factor activity, protein binding
signal transducer activity
Biological Process
Cellular Component
Molecular Function
d
20
18
16
14
12
10
8
6
4
2
0
Precent of DEG
Ribosome
Carbon metabolism
Photosynthesis - antenna proteins
Peroxisome
Endocytosis
Proteasome
Spliceosome
Pentose phosphate pathway
Starch and sucrose metabolism
Biosynthesis of amino acids
Glutathione metabolism
Pyrimidine metabolism
Plant-pathogen interaction
Ubiquitin mediated proteolysis
RNA polymerase
Alanine, aspartate and glutamate metabolism
Glycine, serine and threonine metabolism
Phenylpropanoid biosynthesis
Pentose and glucuronate interconversions
Oxidative phosphorylation
2-Oxocarboxylic acid metabolism
Purine metabolism
Plant hormone signal transduction
Fructose and mannose metabolism
Amino sugar and nucleotide sugar metabolism

Figure S4 The GO and KEGG annotations of DEGs between XJ and ZM.

In the absence of salt stress, the distribution of these GO terms (**a**) and KEGG (**b**) of DEGs between XJ and ZM. Under salt stress conditions, the distribution of these GO terms (**c**) and KEGG (**d**) of DEGs between XJ and ZM. The KEGG pathways pointed by arrows represent the pathways contributing to plant stress-resistance in previous studies.

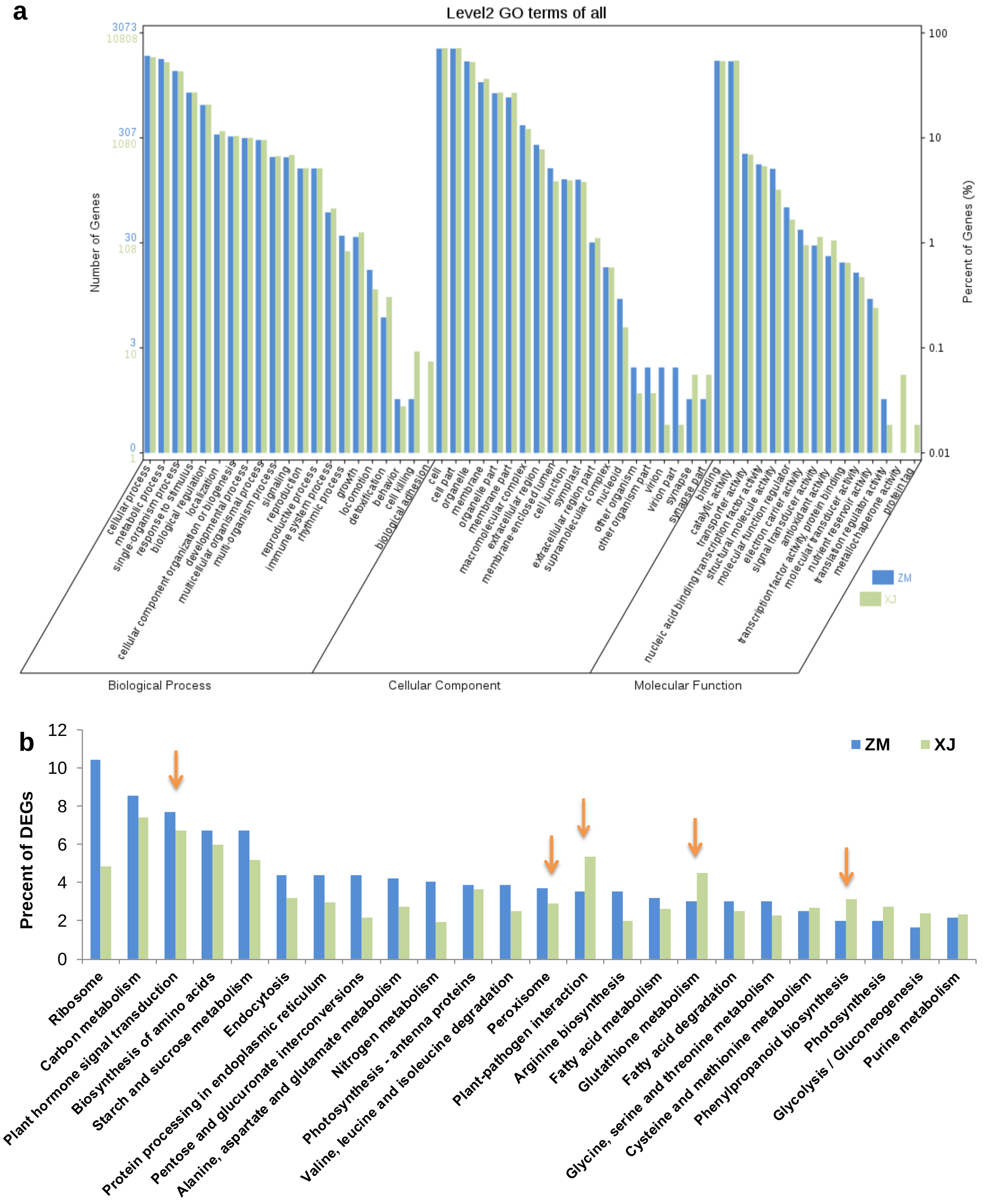

Figure S5 The GO and KEGG annotations of the salt-responsive genes compared between XJ and ZM.

The GO terms (**a**) and KEGG pathways (**b**) of salt-responsive genes compared between XJ and ZM. The KEGG pathways pointed by arrows represent the pathways contributing to plant stress-resistance in previous studies.
